# Reversing the testing effect by feedback is a matter of performance criterion at practice

**DOI:** 10.3758/s13421-020-01041-5

**Published:** 2020-05-16

**Authors:** Mihály Racsmány, Ágnes Szőllősi, Miklós Marián

**Affiliations:** 1 Department of Cognitive Science, Budapest University of Technology and Economics, Egry József utca 1, Budapest, 1111 Hungary; 2 Institute of Cognitive Neuroscience and Psychology, Research Centre for Natural Sciences, Budapest, Hungary

**Keywords:** (Reversed) testing effect, Feedback-based learning, Practice criterion, Cued recall

## Abstract

Retrieval practice is generally considered to be one of the most effective long-term learning strategies and is presumed to be more favorable than repeated study. However, a few recent studies have demonstrated that repetitive feedback at final recall can reverse the long-term advantage of testing over restudy. The result that feedback at long-term tests can dramatically decrease the relative effectiveness of retrieval-based learning could be important for both theoretical and practical reasons. Considering that these earlier studies administered low retrieval success at retrieval practice, we investigated whether the effect of feedback on the testing effect is modulated by the level of retrieval success during practice. In three experiments the level of success at retrieval practice was manipulated by multiple pre-practice learning trials, and multiple tests with feedback were applied after a 1-week retention interval at final recall. Our results have demonstrated that a feedback-induced reversed testing effect was present only at low retrieval success during practice (Experiment 1), whereas with moderate (Experiment 2) and high retrieval success (Experiment 3) during practice a significant testing effect emerged and no reversed testing effect was found even after repeated cycles of feedback. These results point to the conclusion that the level of retrieval success was the key factor in reversing the testing effect in earlier studies. Application of high retrieval success during practice can produce long-lasting accessible memories even in learning settings applying multiple tests with feedback.

## Introduction

Test in the form of retrieval practice can boost long-term learning. An extensive amount of research has shown that taking a memory test on some learning material can improve long-term retention relative to repeatedly studying the material, a phenomenon known as the testing effect (e.g., Carrier & Pashler, 1992; Roediger & Butler, 2011; Roediger & Karpicke, 2006a, 2006b; Wheeler & Roediger, 1992). Knowledge acquired by retrieval practice is more resistant to interference effects and shows a lower forgetting rate (Kliegl & Bauml, 2016; Racsmány & Keresztes, 2015; Szpunar, McDermott, & Roediger, 2008; but see Siler and Benjamin, 2019, for evidence that under certain conditions, testing does not appear to reduce forgetting, but it is a potent means of enhancing inference). Additionally, retrieval practice produces better organization of the acquired knowledge, enhances its transfer to new contexts, and produces faster access to learned information (Jacoby, Wahlheim, & Coane, 2010; Racsmány, Szőllősi, & Bencze, 2018; Zaromb & Roediger, 2010). Altogether these characteristics of retrieval-based learning make test a potential powerful tool for improving learning in everyday educational practice (Dunlosky, Rawson, Marsh, Nathan, & Willingham, 2013; McDermott, Agarwal, D’Antonio, Roediger, & McDaniel, 2014; Roediger, Putnam, & Smith, 2011).

However, two recent studies have presented results that challenge the nimbus of retrieval-practice as one of the most effective learning strategies showing that repetitive feedback at final test can reverse the long-term advantage of testing over repeated study (Pastötter & Bäuml, 2016; Storm, Friedman, Murayama, & Bjork, 2014). Storm et al. (2014) conducted two experiments in which participants were presented with 36 Swahili-English word pairs at encoding in both experiments. In Experiment 1, following this initial study phase, participants took part in a repeated-practice phase where 12 word pairs were restudied, 12 word pairs were tested by cuing the English words with the Swahili words, and the remaining 12 word pairs served as baseline and were not shown during the practice phase. Following a 1-week retention interval participants took part in a delayed final test where all 36 studied word pairs were tested by cuing the English words with the Swahili words. Importantly, immediate feedback was provided after each test trial by presenting the correct English response word to the pariticipants. This process was repeated for a total of six test/feedback sessions, therefore, all 36 word pairs were tested a total of six times. A small but significant testing effect emerged on the first delayed test, as the performance was better in the testing (*M* = 25%) than in the study (*M* = 18%) condition, and recall success in both conditions was significantly better in comparison with the baseline condition (*M* = 5%). The most important result of this experiment was that, following the first test, in all subsequent five delayed tests a reversed testing effect was observed. Specifically, performance was better in the restudy condition than it was in the test condition, and the magnitude of the reversed testing effect increased as a consequence of repeated feedback cycles (Storm et al., 2014, Experiment 1).

Storm and colleagues conducted a second experiment with an almost identical design and procedure to those of Experiment 1, with one important difference: in Experiment 2 participants received repeated feedback during the practice phase for all tested and restudied items. In Experiment 2 no reversed testing effect was detected at the delayed tests. Performance was higher in the testing condition than it was in the restudy condition at the first delayed test, and this advantage of tested items remained significant over all of the six test/feedback cycles (Storm et al., 2014). Storm and colleagues concluded that difficult test practice without feedback could yield long-term advantage over restudy; however, even a single further study opportunity in the form of feedback is sufficient to reverse the testing effect. In contrast, when retrieval practice is combined with additional restudy opportunity in the form of feedback, the long-term advantage of testing over restudy persisted, even after repeated feedback cycles during the final test phase (Experiment 2).

These results may have important theoretical and practical consequences for the literature of retrieval-based learning. The majority of the experiments in this field contrasted the long-term effects of retrieval practice with restudy by applying a relatively low retrieval criterion without feedback at the beginning of the practice phase. According to Storm et al. (2014), these studies may have consistently found a long-term advantage of retrieval practice over restudy because they applied only a single criterion long-term test. Nevertheless, the long-term advantage of retrieval practice over restudy on a single test can hide the disadvantage of testing in promoting long-term retention in a learning environment with feedback-induced restudy. When students have the possibility to check the correct responses in the form of feedback and consequently restudy the learned information after a long retention interval, restudy practice or testing combined with feedback-induced restudy may produce superior long-term performance over pure testing practice.

This line of thinking gained apparent support in Storm et al.’ (2014) experiments, although there is one important aspect to consider. That is, feedback given by providing the correct answers instead of only indicating whether a response was correct or not (e.g., Agarwal, Karpicke, Kang, Roediger, & McDermott, 2008; Butler, Karpicke, & Roediger, 2008; Kang, McDermott, & Roediger, 2007; Pashler, Cepeda, Wixted, & Rohrer, 2005; Storm et al., 2014) serves as an additional study opportunity, given that presenting the cue-response pairs in full allows participants to encode the correct response again (Butler & Roediger, 2008). Arguably, this method of practice, applied in an experimental design, conflates the effects of testing and repeated studying on the retention of the to-be-learned material (Karpicke, Lehman, & Aue, 2014; Roediger & Karpicke, 2006b).

Feedback-induced reversal of the testing effect was also demonstrated by a recent electrophysiological study (Pastötter & Bäuml, 2016). This study applied a markedly different procedure in comparison with the study of Storm and colleagues, presumably to accommodate the specific requirements of the proper EEG analysis. Here participants studied 120 weakly associated word pairs (e.g., linen-TOWEL); the delay between retest/restudy practice and the final test phase was 48 h instead of 1 week, and both the retrieval practice and the final test were different. Specifically, in this study, three letter options were shown and participants were instructed to indicate the last letter of the target choosing from the three options. However, this uncommon testing method inherently allows for the possibility of participants correctly guessing target items instead of retrieving them, even though they were instructed not to guess. The possible conflated effect of guessing in this paradigm renders it difficult to assess real retrieval rates. There was only one feedback cycle after the first test and the reversal of the testing effect was detected on the second test. The detailed analysis of the electrophysiological data of Pastötter and Bäuml’s study (2016) is beyond the scope of the present paper; however, it is important that the reversal of the testing effect by feedback was demonstrated using a quite different experimental procedure.

Certainly, these experiments are not the only ones that have shown that test-based practice, in some circumstances, leads to lower memory retention than restudy. Peterson and Mulligan (2013) have shown that if final free recall primarily benefits from inter-item relationships, then restudy results in greater memory performance than test (see also Mulligan & Peterson, 2013, 2015a, 2015b). The authors termed this phenomenon the negative testing effect and suggest that test is primarily driven by item-specific and cue-target relational processing, so test will be beneficial in those learning situations where item-specific as well as cue-target relational information determines recall. This account is in line with the results of Racsmány et al. (2018), who showed that test-based practice significantly increased the processing speed of cue-item relational processing during test and this change was strongly related to the magnitude of the testing effect. Although the results of experiments investigating the negative testing effect are difficult to compare with the study of Storm and colleagues due to a variety of factors (e.g., short retention delay, low practice rate, and free-recall test format), they raise the possibility that repeated feedback may increase inter-item processing of studied information and thus may reverse the testing effect.

### Theoretical implications of the feedback-induced reversal of the testing effect

The pattern of these previous results is compatible with the distribution-based bifurcation interpretation of the testing effect (Halamish & Bjork, 2011; Kornell, Bjork, & Garcia, 2011). According to this model, during an initial study phase, the to-be-learned items are distributed continuously on a memory-strength dimension, and any opportunity to restudy the items moves this distribution to the right. The bifurcation theory postulates that retrieval practice bifurcates the distribution, and successfully retrieved items are strengthened to a better degree than restudied items. In contrast, items that are not recalled are left with the same memory strength as the unpracticed baseline items. As a consequence, after restudy practice a larger number of items will be above the recall threshold than after test practice; however, successfully recalled items moved further above the recall threshold than restudied items. When the delay between practice and final test is short (a few minutes), the bifurcation theory assumes that more restudied items will be above the recall threshold than tested items. However, when the delay between practice and final test is longer (days), and the distribution of strength moves below the threshold, the bifurcation theory assumes that more tested items will be above the recall threshold than restudied items. In other words, the bifurcation theory of the testing effect is able to explain the so-called test-delay interaction, which is the short-term disadvantage and long-term advantage of test practice in contrast with restudy practice (e.g., Roediger & Karpicke, 2006a; Wheeler & Roediger, 1992). The bifurcation theory accounts for the results of the above-detailed studies (Pastötter & Bäuml, 2016; Storm et al., 2014). As stated by Storm and colleagues: “Although testing may ensure that a larger proportion of items surpass this threshold by providing a substantial boost in the strength of items that are successfully retrieved… it is possible that many of the studies demonstrating long-term benefits of testing without feedback compared with restudying in terms of promoting long-term retention would have also demonstrated significant impairments in terms of its ability to promote accumulations in storage strength across the entire set of to-be-learned information” (Storm et al., 2014, p. 88).

However, the results of feedback-induced reversal of the testing effect may not be compatible with other popular theoretical explanations, such as the semantic elaboration and the episodic context accounts. The former supposes that retrieval in the form of testing of previously learned information prompts elaborative or deep processing of the information (Carpenter, 2009, 2011). This model also assumes that in retrieval-based practice the person recalls words associated with the cue. Later, these semantic associations generated during the test provide an extra retrieval cue, which is why test-based practice is more successful in the long run than the restudy method (Carpenter, 2009, 2011; Pyc & Rawson, 2009). In contrast, the episodic context explanation assumes that the testing effect is due to the fact that the temporal context of the practice is added to the learning context during the retrieval practice (Karpicke et al., 2014). This leads to a complex contextual representation that will, in the long run, be able to significantly narrow down the search for potential target memories and thus enhance memory performance (Karpicke et al., 2014). While these theories are specific to the processes through which the test exerts its long-term beneficial effects, they do not include mechanisms to explain why, in the long term, re-learning based on repeated feedback reverses the testing effect.

### The possible role of performance criterion at practice in feedback-induced reversal of the testing effect

If we take a closer look at the results of Storm et al. (2014), it becomes clear that the difference between their first and second experiments was not only that there was no feedback during practice in Experiment 1, while in Experiment 2 there was, but it might be equally important that in the first experiment, retrieval success was extremely low, while in the second experiment (due to repetitive feedback), it was quite high. It is important to consider Bjork (1975) in this regard: „…an item's state in memory is modified by its retrieval and, more importantly, that the extent of such modification is a function of the depth or level of the retrieval processes” (p. 142). If retrieval practice is preceded by a superficial learning round, the rather low retrieval success rate (ca. 20%) also indicates that most of the retrieval trials consist of superficial cue processing, not followed by reactivation of the experimental context associated with successful retrieval and recollective access to the original learning episode. This is also the case in the experiments of Storm et al. (2014) as well as Pastötter and Bäuml (2016): In the first experiment of Storm et al. (2014), during the practice phase, only 28% of the items could be recalled during the first practice session and after six cycles of practice still not more than 30%. Thus, subjects did not recall 70% of the items in the test condition, even though they encountered the cue words six times during practice.

One important issue is the importance of successful retrieval at practice (for an overview, see Karpicke et al., 2014). Specifically, if initial retrieval success at the beginning of practice is relatively low, testing does not lead to better memory retention than restudy does. Karpicke et al. (2014) introduced a suggestion regarding this issue. Subjects should reach a criterion level, that is, they should be able to retrieve each item at the beginning of practice. Therefore, experiments should be designed to ensure a relatively high retrieval success (see also Karpicke & Roediger, 2007).

The relationship between recall performance during practice and long-term learning is also relevant to another influential theory of memory. Bjork and Bjork (1992) emphasize in the new theory of disuse (NTD) that not only one dimension determines learning success. Long-term learning success is determined by the so-called storage strength, which describes the associative build-up of memory representations, and by the so-called retrieval strength, which depends on the current accessibility of that information. During practice, the change in performance is determined by both storage strength and retrieval strength. Because the model assumes that retrieval strength is completely reset when there is sufficient time and when there is intervening learning between practice and final recall, only storage strength for the tested items can be calculated. This is the main difference between end-of-training performance and long-term performance. For studied items, retrieval strength cannot be measured during the practice phase. However, it was suggested that retrieval strength of the studied items is higher than the retrieval strength of the tested items. That is, without delay, recall performance for the studied items is higher than recall performance for the tested items (e.g., Roediger & Karpicke, 2006a; Wheeler, Ewers, & Buonanno, 2003). NTD also assumes that the successful retrieval of an item will increase storage strength more than re-studying that item. It follows from this notion that if we increase the amount of successful recall it will increase the storage strength, that is, the long-term learning of the tested items. There are two other important statements of the NTD theory. First, the higher the retrieval strength, the smaller the increase in storage strength. We cannot directly measure this in relation to the tested/studied conditions, but based on previous results in the literature, we can assume that retrieval strength of the studied items is higher (see above). So we expect a smaller increase in storage strength for the studied items, as a consequence of pre-practice learning. Second, the higher the storage strength, the more the retrieval strength increases. In sum, in our experiments, we would expect that by increasing the storage strength by increasing the prior learning, retrieval practice leads to a greater increase in storage strength during the practice phase than does repeated study practice.

We hypothesized that the reversal of the testing effect by feedback in earlier studies was due to the extremely low recall success rate during recall practice. Unfortunately, in previous studies, the effects of feedback during practice and recall success cannot be separated, as higher recall success was conflated with re-learning through feedback. Therefore, the primary purpose of the experiments presented here was to increase the success level of practice without the conflated effect of retrieval-based learning and restudy.

In the first experiment, we aimed to replicate the first experiment of Storm et al. (2014) with a completely identical methodology. In the second and third experiments we manipulated one variable. Specifically, we increased the number of initial presentations of the items before practice (retrieval or restudy), which we expected would lead to an increase in the level of success during practice. The purpose of doing so was to show that raising the rate of success during practice alone can ensure that, after a long-term (1-week) delay, even repetitive recall trials with feedback would not reverse the testing effect.

## Materials and methods

### Participants

Subjects were 84 Hungarian undergraduate students who received either money or extra course credit for participation. Participants had no history of psychiatric/neurological disorders. They gave written informed consent. The study was approved by the United Ethical Review Committee for Research in Psychology, Hungary.

We used G-Power (Version 3.1.9.2; Faul, Erdfelder, Lang, & Buchner, 2007) to calculate required sample size for Experiments 1 and 2. We focused on the critical comparison, specifically, on the difference between the restudy and test conditions on the first final test of the memory task. We used the effect size value (*d* = 0.55) reported in Storm et al. (2014). Additional input values were an alpha error probability of .05 and a power of .80. Based on these parameters, the required sample size was *n* = 28. Expecting some drop-out, we collected data from 30 participants in Experiment 1 and in Experiment 2. One participant was excluded from the sample of Experiment 1, because this subject gave no response at all in the practice phase. The final sample size was, therefore, *n* = 29 in Experiment 1 (four male participants; *M*_*age*_ = 21.5 years, *SD* = 2.1). No participant was excluded from the sample of Experiment 2 resulting in a final sample size of *n* = 30 (seven male participants; *M*_*age*_ = 22.3 years, *SD* = 2.5).

We calculated the required sample size for Experiment 3 on the basis of data of Experiment 2. We used the effect size value for the critical comparison between the study and retest conditions on the first final test in Experiment 2 (*d* = 1.33). Required sample size was *n* = 7; this seemed extremely low, therefore, we collected data from 24 participants as Storm et al. (2014) did in their first experiment (six male participants; *M*_*age*_ = 21.5 years, *SD* = 1.8). No participant was excluded from the sample of Experiment 3.

### Experimental design and procedure

**Fig. 1:**
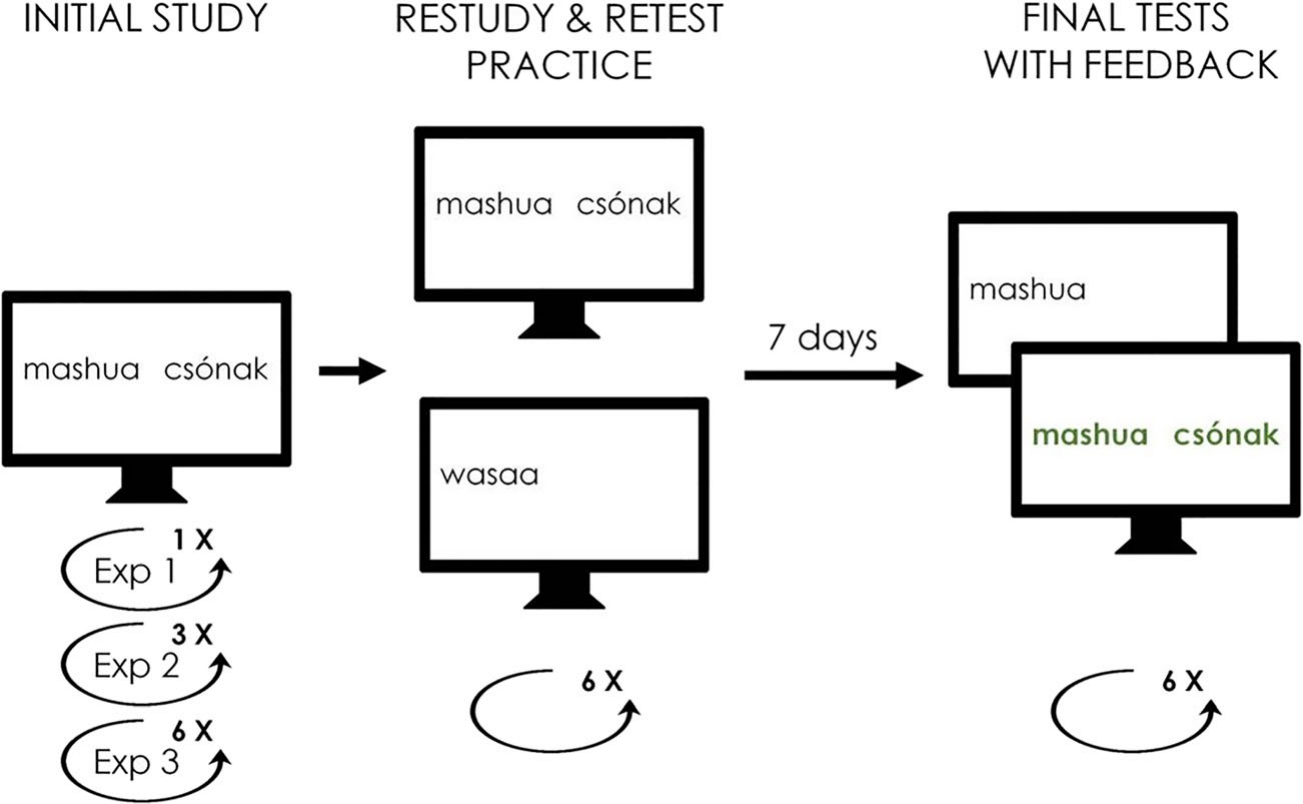
**T**he procedure of the memory tasks. Participants were presented with Swahili-Hungarian word pairs either once (Experiment 1) or three (Experiment 2) or six times (Experiment 3). Word pairs were then practiced in six cycles by either restudy or cued recall (test condition). Following a 1-week retention interval, all word pairs were tested in six cycles, and feedback was given following each trial in the form of re-presenting the word pairs

Stimuli were 36 Swahili-Hungarian word pairs translated from Nelson and Dunlosky (1994). The Swahili and the Hungarian words were randomly paired for each participant. The task (in each experiment) consisted of three phases: initial study, practice, and a delayed final test phase. The procedure is illustrated in Fig. 1.

#### Initial study

In the study phase, participants were presented with all 36 word pairs with the Swahili and the Hungarian words on the left and right side of the screen, respectively (12 s/word pair, pre-stimulus interval (PSI): 0.5 s). Subjects saw one word pair at a time, and were instructed to memorise them. While subjects saw the 36 word pairs only once in Experiment 1, participants were presented with the stimuli in three and six consecutive cycles in Experiments 2 and 3, respectively. There was no delay between the learning cycles in Experiments 2 and 3.

#### Practice

Immediately after the initial study phase, a practice phase followed, which consisted of six practice sessions. The practice phases were identical in Experiments 1, 2, and 3. The word pairs were randomly assigned into one of three conditions: restudy (12 word pairs), test (12 word pairs), and baseline (12 word pairs). The baseline items were not presented at all in this phase. In the restudy condition, word pairs were shown on the screen for 4 s each with a PSI of 0.5 s. Subjects were required to *read* and say out loud the Hungarian word. In the test condition, the Swahili cues were presented on the left side of the computer screen (for 4 s each with a PSI of 0.5 s), and subjects were required to *recall* and say out loud the Hungarian word. The experimenter recorded the responses. Trial types were intermixed with the constraint that a maximum of three consecutive trials included stimuli from the same condition. (This constraint was used in the final test phase as well.) Participants were presented with the study material in a different random order in each practice cycle. Participants did not receive feedback during practice. The practice sessions were separated by 1-min arithmetic distractor tasks consisting of single-digit additions and subtractions that were also shown on the computer screen.

#### Delayed final tests

Following a 7-day retention interval, participants underwent a final test consisting of six test sessions (with no delay between the sessions). Final tests were identical in Experiments 1, 2, and 3. In each final test session, participants’ memory for all 36 word pairs was tested. As in the test condition of the practice phase, the Swahili words were presented with the participants (4 s/cue), and subjects were asked to recall and say out loud the Hungarian equivalents while the experimenter recorded their responses. After each trial, participants received feedback in the form of the word pair being presented in green font for 2 s. Cue presentation and feedback were both preceded by 0.5-s PSIs. Participants were presented with the stimuli in a different random order on each final test.

### Data analysis

For each of the three experiments, we conducted a repeated-measures analysis of variance (ANOVA) on recall success with the six test practice trials as six levels. To analyse final recall success, we conducted 6 × 3 ANOVAs with Test Trial (1–6) and Condition (baseline, restudy, and test) as within-subjects factors. During post hoc analyses, we compared performance between the conditions by conducting a series of paired-samples *t*-tests (restudy vs. baseline, test vs. baseline, and restudy vs. test).

## Results

### Test practice: Experiments 1–3

As a consequence of the relatively large number of study cycles, participants showed better memory performance during test practice in Experiments 2 and 3 than subjects did in Experiment 1 (see Table 1). Recall success improved during the test practice trials in all three experiments, Experiment 1: *F*(5, 140) = 3.15, *p* = .01, η^2^_p =_ .10, Experiment 2: *F*(5, 145) = 12.57, *p* < .001, η^2^_p =_ .30, Experiment 3: *F*(5, 110) = 4.20, *p* < .01, η^2^_p =_ .16.

**Table 1:** Recall success during the six initial test practice trials in Experiments 1, 2, and 3

Experiments	Trial 1	Trial 2	Trial 3	Trial 4	Trial 5	Trial 6
Experiment 1	25.3 (3.8)	25.6 (3.6)	26.1 (3.5)	27.6 (3.7)	27.6 (3.9)	28.7 (3.9)
Experiment 2	66.1 (4.6)	68.6 (4.5)	69.7 (4.5)	71.4 (4.6)	75.0 (4.2)	73.1 (4.5)
Experiment 3	76.1 (4.6)	78.1 (4.4)	76.7 (4.9)	78.8 (4.8)	80.9 (4.6)	80.2 (4.7)

*Notes.* Values represent the means (%); standard errors of the means are shown in parentheses

### Final tests: Experiment 1

**Fig. 2:**
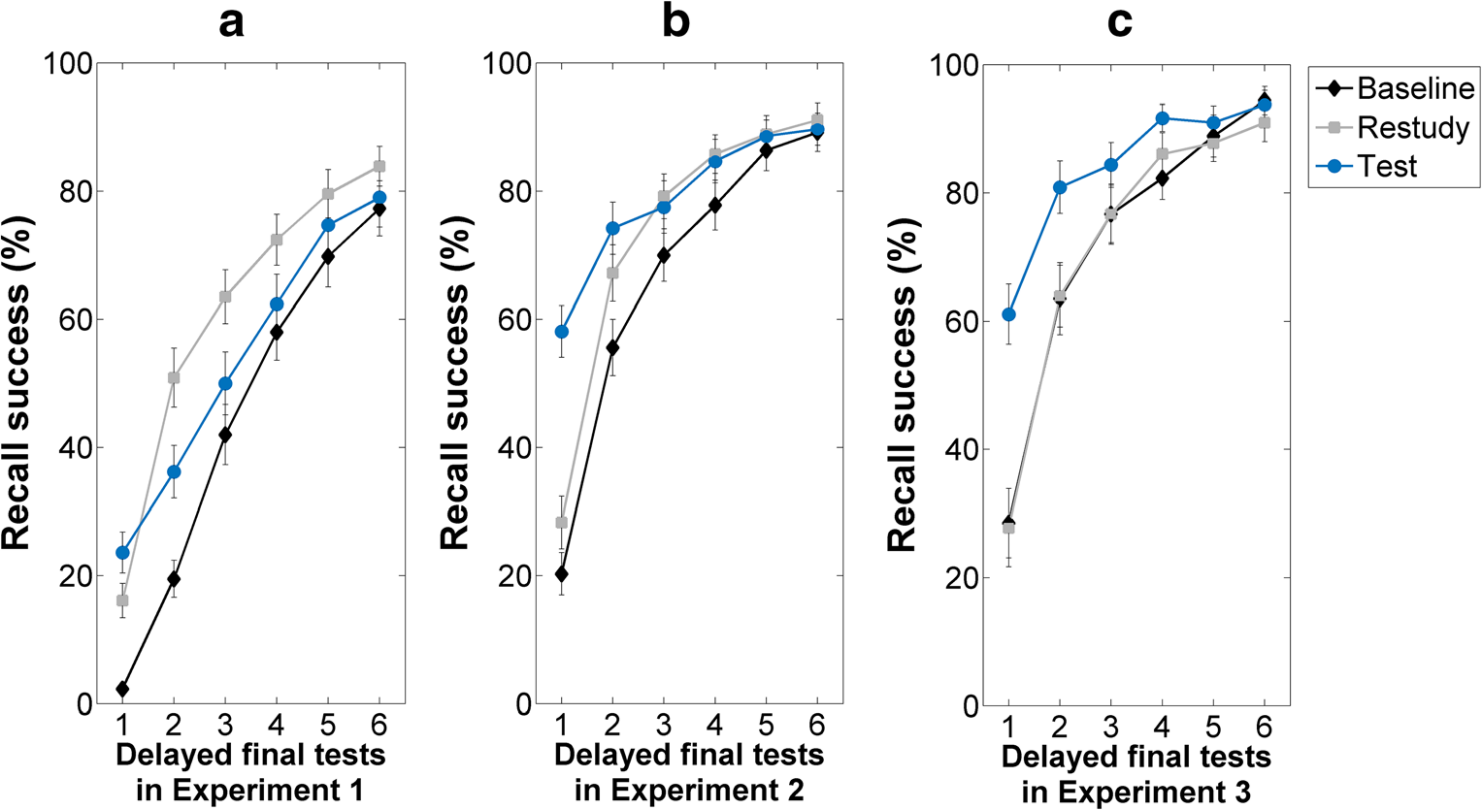
**R**ecall success on the six delayed final tests in Experiment 1 (**A**), Experiment 2 (**B**), and Experiment 3 (**C**). Participants received feedback after each final test trial in all three experiments (in the practice phase subjects were given no feedback). When participants were presented with the study material only once in the initial study phase, the testing effect was reversed after one final test cycle (Experiment 1, **A**). When participants were presented with the study material either three times (Experiment 2, **B**) or six times (Experiment 3, **C**) in the initial study phase, no reversed testing effect was found on the delayed final tests. Instead, participants’ memory was better for the tested items than it was for the restudied word pairs on final test 1–4 in Experiment 3 – indicating significant testing effects. Error bars represent the standard errors of the means

Recall success improved during the final test trials in Experiment 1, as indicated by the main effect of Test Trial, *F*(5, 140) = 266.51, *p* < .001, η^2^_p =_ .91 (see Fig. 2a). The main effect of Condition, *F*(2, 56) = 21.48, *p* < .001, η^2^_p =_ .43, and the Test Trial × Condition interaction, *F*(10, 280) = 8.85, *p* < .001, η^2^_p =_ .24, were also significant.

#### First final test

Recall rate on the first delayed final test was lower in the baseline condition than it was in the restudy, *t*(28) = 5.32, *p* < .001, *d* = 0.99, and test conditions, *t*(28) = 6.35, *p* < .001, *d* = 1.49. Additionally, we found a significant difference between the restudy and test conditions, *t*(28) = 2.29, *p* = .03, *d* = 0.43, with relatively better memory performance for the tested items.

#### Subsequent final tests

In each subsequent final test block (i.e., on final test 2**–**6) memory was better for the restudied word pairs than it was for the baseline items, all *t*s ≥ 2.11, all *p*s < .05, all *d*s ≥ 0.39. Recall rates differed between the baseline and test conditions (with better memory for the tested items) only in the second final test block, *t*(28) = 4.51, *p* < .001, *d* = 0.84. Most importantly, participants showed better memory for the restudied items than they did for the tested items on final tests 2, 3, and 4, all *t*s ≥ 2.63, all *p*s < .05, all *d*s ≥ 0.49. These latter results indicate that the testing effect was reversed after one single final test session.

### Final tests: Experiment 2

As in Experiment 1, the ANOVA indicated significant main effects of Test Trial, *F*(5, 145) = 205.99, *p* < .001, η^2^_p =_ .88, and Condition, *F*(5, 58) = 27.23, *p* < .001, η^2^_p =_ .48, as well as a significant Test Trial × Condition interaction, *F*(10, 290) = 27.81, *p* < .001, η^2^_p =_ .49 (see Fig. 2b).

#### First final test

Recall success for the baseline items was worse than it was for the restudy, *t*(29) = 2.55, *p* < .05, *d* = 0.47, and tested items, *t*(29) = 11.75, *p* < .001 *d* = 2.15. Additionally, a strong testing effect was found, as indicated by better memory for the tested word pairs, when compared to the restudy condition, *t*(29) = 7.27, *p* < .001 *d* = 1.33.

#### Subsequent final tests

Recall success was better for the restudied word pairs than it was for the baseline items on final tests 2, 3, and 4, *t*s ≥ 3.39, all *p*s ≤ .01, all *d*s ≥ 0.62. When we compared recall success between the baseline and test conditions, a significant difference was found on final tests 2, 3, and 4, *t*s ≥ 3.73, all *p*s ≤ .01, all *d*s ≥ 0.68, with better memory for the tested word pairs.

Most importantly, no reversed testing effect was found. While on final test 2 recall rate was higher for the tested items than it was for the restudied items, *t*(29) = 2.89, *p* < .01, *d* = 0.53, recall rates did not differ between the restudy and test conditions on final tests 3, 4, 5, and 6, all *p*s > .05.

### Final tests: Experiment 3

Just as in the first two experiments, Test Trial, *F*(5, 115) = 126.88, *p* < .001, η^2^_p =_ .85, and Condition, *F*(2, 46) = 22.81, *p* < .001, η^2^_p =_ .50, had main effects on recall success, and the interaction between these variables, *F*(10, 230) = 13.73, *p* < .001, η^2^_p =_ .37, was also significant (see Fig. 2c).

#### First final test

Although recall rate for the tested items was higher than it was for the baseline items, *t*(23) = 7.37, *p* < .001, *d* = 1.50, we found no significant difference between the restudy and baseline conditions, *t*(23) = 0.17, *p* = .86, *d* = 0.04. And, again, a strong testing effect was found, *t*(23) = 8.37, *p* < .001, *d* = 1.71.

#### Subsequent final tests

On the subsequent final tests, memory performance did not differ between the baseline and restudy conditions, all *p*s > .05, whereas recall rate was higher for the tested items than it was for the baseline items on final tests 1, 2, and 3, all *t*s ≥ 2.04, all *p*s < .05, all *d*s ≥ 0.42.

Finally, and importantly, no reversed testing effect was found. Instead, subjects showed better memory for the tested word pairs than they did for the restudied items on final tests 2, 3, and 4, all *t*s ≥ 2.28, all *p*s < .05, all *d*s ≥ 0.46. On final tests 5 and 6 there was no significant difference between the restudy and test conditions, both *p*s > .05.

## General discussion

In conclusion, our first experiment replicated the results of the first experiment of Storm et al. (2014). Following one initial presentation of the word pairs, subjects were able to recall less than 30% of the learned items during the six training sessions (see Table 1). After a 1-week delay, in the first round of final recall, previously tested items were recalled better than previously restudied items. However, after re-learning with a single feedback, the testing effect was reversed and the restudied items were better recalled during the second recall attempt. The reversal of the testing effect was present at each further learning round. Similar to Storm et al.’ (2014) first experiment, the recall of the tested items on the fourth recall did not differ from the baseline items that had not been practiced before (see Fig. 1).

The second experiment involved a single modification to the first experiment. Here, we presented the to-be-learned word pairs three times before starting practice, assuming that this will result in a higher success rate during retrieval practice. This was the case, and the success rate in the practice phase ranged between 66% and 73%. After a 1-week delay, a significant testing effect was observed on the first recall test. Even after five cycles of recall this testing effect did not reverse. In fact, in the second test round, after the first feedback, a significant testing effect was still found, as the tested items were better recalled than the restudied items. After the second feedback, there was no difference in the recall of the tested and restudied items, but the recall rate of the restudied words never exceeded that of the tested items, even after the fifth feedback, although performance was near ceiling (see Fig. 1). The third experiment yielded an even more robust result. Here we presented the to-be-learned word pairs six times before practice. As a result, the success rate was very high during practice. Subjects recalled 76–80% of the items tested during the six practice sessions. After a 1-week delay, a robust testing effect was found, and the effect remained even after three feedbacks. Subjects recalled significantly more tested items than restudied items even in the fourth recall cycle. Memory for the restudied items only reached memory for the tested items in the last two trials, but the performance was already in the ceiling zone. In summary, therefore, simply by increasing the average recall success rate, without re-learning through feedback during retrieval practice (as in Experiment 2, Storm et al., 2014), the testing effect after a delay of 1 week proved to be resistant to multiple cycles of feedback-induced relearning.

**Fig. 3:**
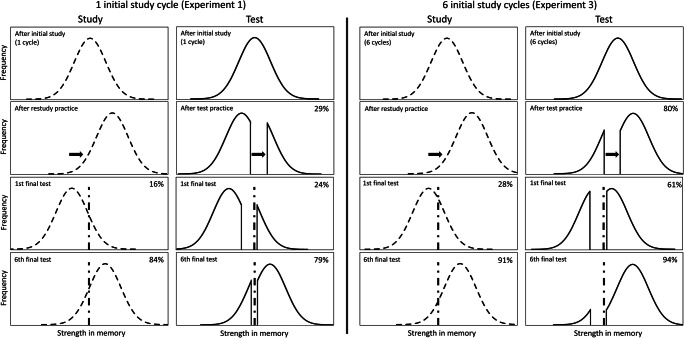
**I**nterpreting results from Experiments 1 and 3 in the bifurcation framework (Kornell et al., 2011). In Experiment 1, after one initial study cycle (just as in Storm et al.’ (2014) Experiment 1), repeated study practice moves the distribution of all restudied items to the right, whereas retest practice only strengthens items that are recalled, causing a bifurcated distribution. During the 7-day retention period, memory strength of all items decreases, resulting in a relatively low performance on the first final test for restudied items (16% recalled), and a significantly better, although still low, performance for retested items (23%), as a result of the previously bifurcated distribution. However, giving feedback after each retrieval attempt during the final test cycles boosts memory strength for all items, but because of the “gap” the bifurcation caused for retested items, these items receive less strengthening altogether. Altogether this results in restudied items being recalled to a better degree than retested items after feedback, and the testing effect reverses. Importantly, in Experiment 3, the initial memory strength of all items is higher due to the six (as opposed to one) initial learning cycles; the distributions are more to the right. Just as in Experiment 1, restudy practice strengthens all items and retest practice bifurcates the distribution. However, bifurcation, thanks to the higher initial strength of memories, occurs at a different point of the distribution, so more items are recalled in retest practice phase than in that of Experiment 1 (80% vs. 29%). This better performance in the practice phase in turn leads to better performance on the first final test, where a large testing effect is observed (61% of retested and 28% of restudied items recalled). Even though the distribution of retested items could still be considered bifurcated, the testing effect persists even after multiple test and feedback cycles. This suggests that boosting the initial memory strength of items to a sufficiently high level prevents the reversal of the testing effect even after multiple feedbacks

Storm et al.’ (2014) interpretation of the findings of their second experiment was that retrieval with feedback during practice exerts a different effect on memory strength than retrieval without feedback, and, therefore, there is no reversal of the testing effect. However, we do not consider this assumption to be necessary. As shown in Fig. 3, the bifurcation model (Kornell et al., 2011) can describe the difference between our first and third experiments (and thus the difference between our first and second experiments). Accordingly, by increasing recall success in the practice phase in Experiment 3 with multiple initial item presentations, the strength of memory trace is continued to bifurcate for tested items, with only a change in the proportion of items above and below the threshold. As illustrated in Fig. 3, by moving the bifurcation point to the right it can be predicted that in the long run the first recall and the subsequent recalls modified by feedback retain the testing effect. In contrast to Storm et al.'s (2014) second experiment, we did not achieve this by conflating retrieval practice with restudying during practice. So, here, we achieved through pure recall practice that the testing effect persisted in the long run, even after multiple feedbacks. That is, as can be interpreted within the bifurcation model (Fig. 3), in earlier experiments the reversal of the testing effect was due to the success rate of practice being too low, and the bifurcation point being moved too far to the left.

Recently, Kliegl, Bjork, and Bäuml (2019) investigated the effect of feedback given on the final tests after retest practice in two conditions (easy vs. difficult recall during practice). On the first final test recall success was better in the difficult recall condition, but this effect was reversed as a result of feedback. Although it is difficult to compare the findings of this experiment and the results of the present study due to several methodological differences, the results point to one direction that experimental manipulations that increase performance during test practice also increase long-term feedback-related improvement.

These results also fit well with the hypotheses based on the NTD (Bjork & Bjork, 1992). The model assumes that during practice, the change in performance is determined jointly by storage strength and retrieval strength. Hence, we can assume that if we increase the amount of successful recall it will increase the storage strength, that is, the long-term learning of the tested items. As the difference between performance during practice and performance during the final test may be a good indicator of storage strength for the tested items, the model would assume that this number will be lower as a result of the increased number of preliminary presentation rounds. This is exactly what we find, which suggests that the increase in preliminary presentation rate resulted in greater storage strength during retrieval practice in Experiments 2 and 3 than in Experiment 1. Furthermore, in accordance with the assumptions of the NTD, the increase of the preliminary presentation led to a very robust increased testing effect, as demonstrated in Experiments 2 and 3.

In summary, our results support the bifurcation model and also can be explained by the semantic elaboration and the episodic context theories. As a result of the test, the set of items did separate. For those items that can be successfully recalled during practice, later on after a long delay, feedback-based learning results in reactivation, whereas for those that are never recalled during practice, a new learning process begins, similar to the baseline items. Conversely, for restudied items, reinforcement of memory strength is evenly distributed from very weak to strong, but some level of learning occurs for each item. Our results also support the episodic context theory (Karpicke et al., 2014): without successful retrieval and context reactivation there is no long-term improvement. Unsuccessful retrieval attempts could not relate the context of the practice to the information to be learned. This is similarly understood from the point of the semantic elaboration theory (Carpenter, 2009), even though the participants generate semantically associated information to the cue during practice, in most cases this is not related to the target memory, as the retrieval is unsuccessful.

Our results also have important consequences for education practice. For long-term success of retrieval-based practice, it is necessary to achieve a fairly high level of retrieval success during the practice. If practice comes at a time when retrieval is difficult but still successful (Bjork, 1975), the information acquired through the test is not only more resistant to forgetting than restudied information, but feedback will also result in greater performance gains.

## Data Availability

The datasets generated and analyzed during the current study are available in the Open Science Framework (OSF) repository, https://osf.io/ucxj2/.
